# A Review of the Giant Protein Titin in Clinical Molecular Diagnostics of Cardiomyopathies

**DOI:** 10.3389/fcvm.2016.00021

**Published:** 2016-07-21

**Authors:** Marta Gigli, Rene L. Begay, Gaetano Morea, Sharon L. Graw, Gianfranco Sinagra, Matthew R. G. Taylor, Henk Granzier, Luisa Mestroni

**Affiliations:** ^1^Adult Medical Genetics Program, Cardiovascular Institute, University of Colorado Denver, Aurora, CO, USA; ^2^Department of Cardiology, Hospital and University of Trieste, Trieste, Italy; ^3^Molecular Cardiovascular Research Program, University of Arizona, Tucson, AZ, USA

**Keywords:** titin, *TTN*, familial cardiomyopathy, cardiovascular genetics, clinical genetics, heart failure, clinical diagnosis

## Abstract

Titin (*TTN*) is known as the largest sarcomeric protein that resides within the heart muscle. Due to alternative splicing of *TTN*, the heart expresses two major isoforms (N2B and N2BA) that incorporate four distinct regions termed the Z-line, I-band, A-band, and M-line. Next-generation sequencing allows a large number of genes to be sequenced simultaneously and provides the opportunity to easily analyze giant genes such as *TTN*. Mutations in the *TTN* gene can cause cardiomyopathies, in particular dilated cardiomyopathy (DCM). DCM is the most common form of cardiomyopathy, and it is characterized by systolic dysfunction and dilation of the left ventricle. *TTN* truncating variants have been described as the most common cause of DCM, while the real impact of *TTN* missense variants in the pathogenesis of DCM is still unclear. In a recent population screening study, rare missense variants potentially pathogenic based on bioinformatic filtering represented only 12.6% of the several hundred rare *TTN* missense variants found, suggesting that missense variants are very common in *TTN* and are frequently benign. The aim of this review is to understand the clinical role of *TTN* mutations in DCM and in other cardiomyopathies. Whereas *TTN* truncations are common in DCM, there is evidence that *TTN* truncations are rare in the hypertrophic cardiomyopathy (HCM) phenotype. Furthermore, *TTN* mutations can also cause arrhythmogenic right ventricular cardiomyopathy (ARVC) with distinct clinical features and outcomes. Finally, the identification of a rare *TTN* missense variant cosegregating with the restrictive cardiomyopathy (RCM) phenotype suggests that *TTN* is a novel disease-causing gene in this disease. Clinical diagnostic testing is currently able to analyze over 100 cardiomyopathy genes, including *TTN*; however, the size and presence of extensive genetic variation in *TTN* presents clinical challenges in determining significant disease-causing mutations. This review discusses the current knowledge of *TTN* genetic variations in cardiomyopathies and the impact of the diagnosis of *TTN* pathogenic mutations in the clinical setting.

## Introduction

Dilated cardiomyopathy (DCM) is defined by the presence of left ventricular (LV) or biventricular dilatation and systolic dysfunction in the absence of hypertension, valvular disease, or coronary artery disease sufficient to cause global systolic impairment (1). The prevalence of the disease is about 1:2,500, and DCM explains about half of the heart failure cases in the United States. About 35–40% of DCM cases are classified as “idiopathic” or “familial/genetic” cardiomyopathy (2). Other causes of the DCM phenotype are ischemic, congenital, valvular, inflammatory, or cardiotoxic heart disease. Finally, other rare cardiomyopathies, such as hypertrophic cardiomyopathy (HCM), arrhythmogenic right ventricular cardiomyopathy (ARVC), and restrictive cardiomyopathy (RCM), have genetic causes.

In this setting, genetics can justify a significant proportion of DCM cases (up to 25%), so the disease can be classified into genetic and non-genetic forms (3). In DCM, the most common form of cardiomyopathy, more than 50 genes have been associated with the phenotype, usually with incomplete penetrance and variable expressivity, and frequently with familial transmission (2–4). Evidence suggests that familial DCM is inherited in an autosomal dominant pattern in about 90% of cases, but few cases follow an autosomal recessive, x-linked, or mitochondrial pattern of inheritance (5–7). Genes most frequently involved in the disease are encoding structural proteins of the sarcomere (titin and myosin heavy chain), cytoskeleton (desmin), nuclear membrane (lamin A/C), membrane proteins and ion channels (phospholamban and presenilin), protein of the dystrophin-glycoprotein complex (dystrophin and sarcoglycan), desmosomes (desmoplakin and desmoglein), mitochondrial proteins (frataxin), and extracellular matrix proteins (alpha-laminin) (8).

Titin (*TTN*) encodes the largest human protein, whose name stems from the word Titans, giants of Greek mythology. Among the genes involved in cardiomyopathies, *TTN* plays a central role because of its frequency and the key structural, mechanical, and regulatory role within the sarcomere in the striated muscle (9). The *TTN* gene consists of 364 exons, located on chromosome 2q31, that produces maximally a 4,200-kDa protein which is composed of ~38,000 amino acid residues. The size and complex structure of the TTN protein provides architectural support, maintaining the sarcomeric organization during contraction, and developing passive tension during muscle stretching. It also has a sensory and signaling role through the multiple TTN-binding proteins that are organized in signaling hot spots (10–12). The protein is organized in four structural and functional regions: the N-terminal Z-line (anchor to the sarcomeric Z-disk), the I-band (responsible for elastic properties), A-band regions (with a stabilizer role of the thick filament), and the C-terminal M-line extremity (overlap in antiparallel orientation with another C-terminal TTN molecule; modulation of TTN expression and turnover with the tyrosine kinase domain) (10).

Truncation mutations of *TTN* are the most frequent in DCM where 25% of cases are familial forms and 18% are sporadic forms of DCM (13). However, it remains to be confirmed that *TTN* truncating mutations are always pathogenic (3, 14). Interestingly, truncations in the A-band region of *TTN* accounts for up to 25% of DCM cases (15). Furthermore, *TTN* is involved in the pathogenesis of other cardiomyopathies such as HCM and ARVC that is considered to be a genetic disease (30–50% of cases are familial), and RCM.

After the introduction of next-generation sequencing (NGS), the study of *TTN* gene mutations, previously difficult to analyze due to its size and complexity, has now allowed the identification of more than 60,000 *TTN* missense variants (reported in the 1000 Genomes Project) (16, 17). The aim of this review is to discuss the challenges in diagnosing the correlation between *TTN* mutations and the different types of cardiomyopathy in the clinical setting.

## Mechanistic Studies of TTN

**Figure 1:**
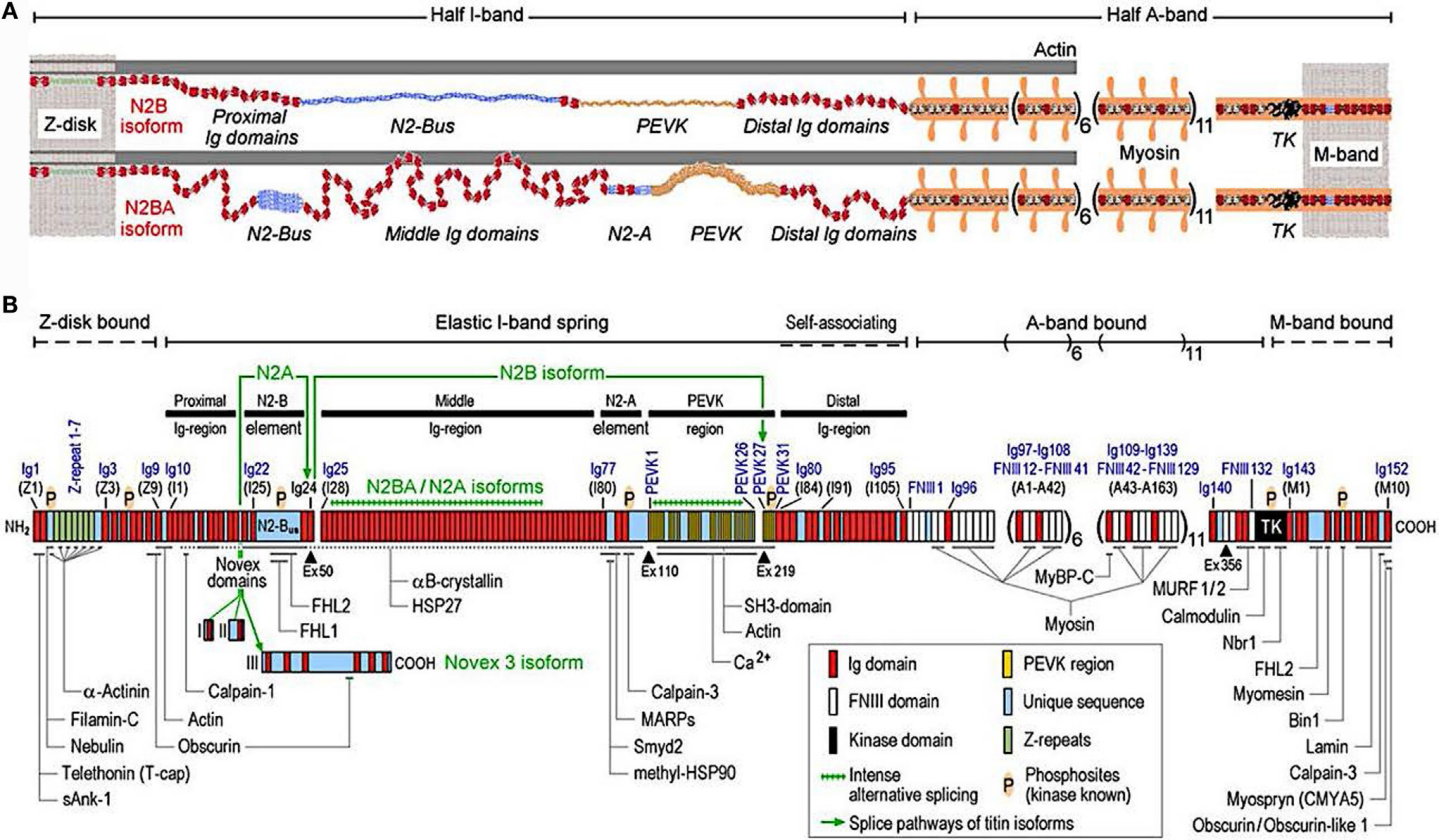
**Domain structure of titin isoforms and binding sites of titin ligands**. **(A)** N2B and N2BA titin isoforms represented in the cardiac half-sarcomere, **(B)** Domain structure of titin sequence, Q8WZ42-1, with ligand binding sites represented (from Linke and Hamdani, with permission) (60).

**Figure 2:**
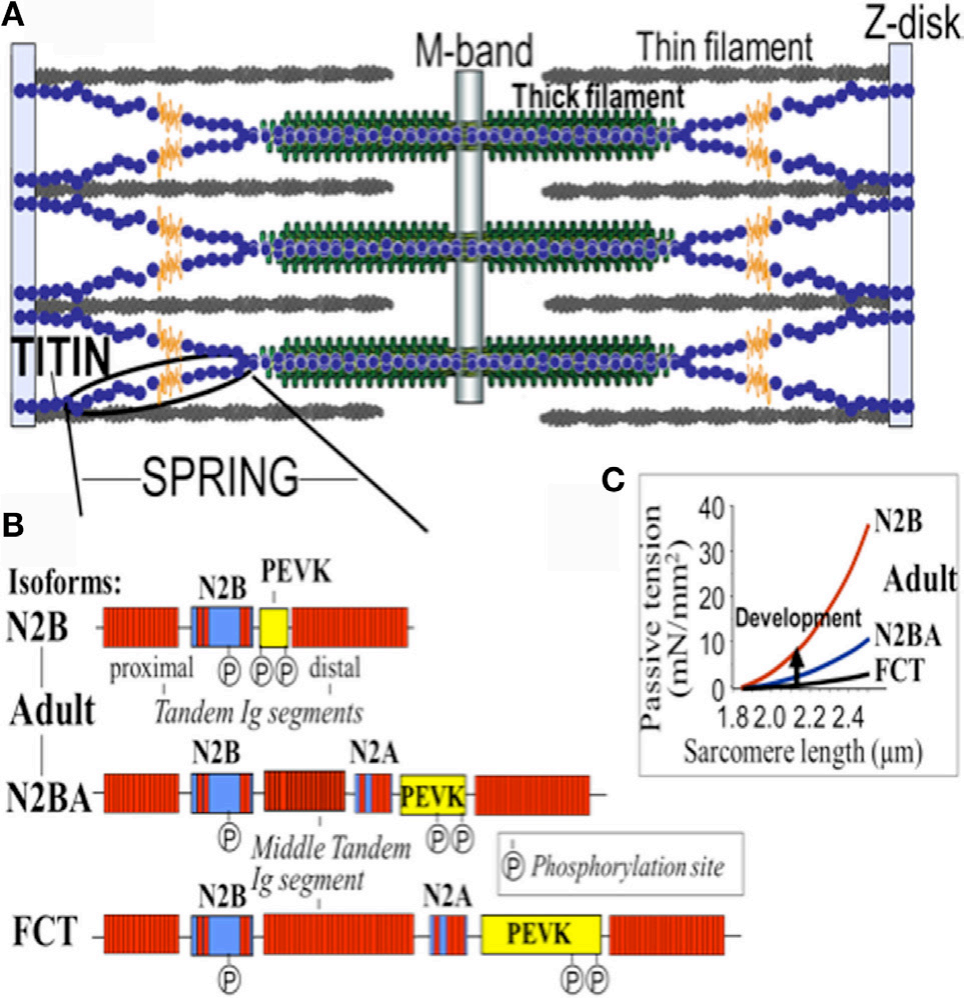
**Domain structure of titin isoforms**. **(A)** The spring segment, **(B)** difference in the domain structure of different isoforms, and **(C)** the relationship of the passive tension with the sarcomere length in the different isoforms. FTC, fetal cardiac titin.

Titin is the largest human protein. Two TTN filaments with opposite polarity span each sarcomere, namely, the contractile unit in striated muscle cells. TTN is responsible for sarcomere passive stiffness generation (18). TTN is composed of a Z-disk at its N-terminus, whereas the remaining part of the molecule is composed of the elastic I-band region (consisting of tandem Ig segments of serially-linked Ig-like domains), the spring-like PEVK region (is composed of proline (P), glutamate (E), valine (V), and lysine (K)), three unique sequences of Novex1, 2, and 3, cardiac-specific N2B and N2A domains, a thick A-band region, and a M-band region embedding the C-terminus (Figures 1 and 2) (19–21). The extensible I-band region gradually lengthens and develops passive tension when the sarcomere is stretched during diastole (15). The inextensible A-band binds myosin and myosin-binding protein C (MyBP-C), whereas the M-band contains a kinase that affects gene expression and cardiac remodeling (22).

The 364 exons of *TTN* undergo extensive alternative splicing to encode different isoforms. In cardiomyocytes, three different isoforms of titin are expressed: adult N2BA, adult N2B, and the fetal cardiac titin (FCT) isoforms. The I-band sequence defines the different proprieties of each isoform, whereas the Z-disk, A-band, and M-line regions are extremely conserved (22). The isoforms, N2BA and N2B are expressed 30–40 and 60–70% respectively, within the TTN protein in healthy adult human heart. The ratio between these two isoforms is a major determinant of the cardiomyocyte stiffness (18). Due to the longer extensible I-band region, the N2BA titin isoform is more compliant than N2B titin (23–25). The compliant N2BA contains additional spring elements in the PEVK and tandem Ig regions and is therefore associated with low cardiomyocyte passive tension (25). The TTN-based passive tension is established by the TTN expression ratio in the human heart. There is a strong relationship between the TTN-based passive tension and the size of the I-band region: the larger the elastic I-band region and the lower the passive tension (22). Variable isoform expression and *TTN* splicing have become of great importance in different cardiac diseases, including DCM, whereby the compliant N2BA isoform is upregulated and is associated with decreasing passive stiffness and increasing chamber compliance (23, 24, 26, 27).

A recent study by Roberts et al. suggested that the clinical significance of *TTN* truncating variants is largely predicated by the exon usage and variant location (the distance of the truncating variant from the protein N-terminus) (28). Furthermore, the authors compared *TTN* truncating variants among different isoforms and found *TTN* truncating variants altering both N2BA and N2B were overrepresented in DCM patients versus controls and more strongly associated with DCM as compared with the *TTN* truncations involving the N2BA isoform only. Conversely, the *TTN* truncations of the controls were composed of exons not incorporated into N2BA and N2B transcripts (28).

The *TTN* gene structure is organized to accommodate extensive splicing events. Roberts et al. defined a percentage spliced in (PSI) score based on RNA sequencing data from end-stage DCM and donor heart in order to find the mean usage of each *TTN* exon (28). The PSI estimates the proportion of transcripts that incorporate a given exon. A high PSI was given to an exon constitutively expressed and present in all *TTN* isoforms, while a low PSI was usually present only in one isoform and had a lower expression. Moreover, exon symmetry was related to PSI: only 3 exons among the 175 with PSI < 0.99 were asymmetric versus 27% of those with PSI > 0.99. Interestingly, the authors found that more than 80% of all *TTN* exons were symmetric and that their exclusion would not alter the translational reading frame. For instance, in the I-band, the region with the lower PSI, 93% of alternately spliced exons were symmetric: a truncating variant in that region will fall in exons spliced out or not expressed in the majority of the transcripts and should not have such a deleterious effect. While the stiffness of TTN is defined primarily by the I-band segment sequence of each isoform, it is well known that the cardiac passive tension can be affected by multiple post-translational modifications of contractile and regulatory proteins (29). Few studies have discovered that protein kinase phosphorylation significantly alters the stiffness of N2B and PEVK spring elements (30, 31). The N2B spring element is phosphorylated by PKA and PKG with a reduction in passive tension (29, 32).

The mechanisms responsible for the changes in *TTN* isoform expression are still not completely understood; however, it has been shown that RNA-Binding Motif Protein 20 (RBM20), a RNA splicing factor, plays an important role in this process and a reduced expression of RBM20 can alter *TTN* splicing and isoform expression in human (33) and mice (34), leading to DCM.

Therefore, the TTN-based myocardial stiffness is determined by the TTN isoform composition and the phosphorylation state of TTN’s elastic I-band. Different kinases can modify the TTN elasticity in different ways; indeed, it is known that changes in post-translational modification (in particular hypophosphorylation) plays a role in the pathophysiology of heart disease (13).

## Titin in the Pathogenesis of Dilated Cardiomyopathy

Dilated cardiomyopathy is a primary myocardial disease with variable natural history and clinical presentation affecting young individuals with a potential long life expectancy. A genetic etiology is demonstrated in ~30% of cases (35), and the giant muscle TTN protein has been recognized as the major human disease-causing gene for DCM (9). The advances in contemporary DNA sequencing and the introduction of NGS have allowed the screening of *TTN* in large cohorts of patients with DCM and in the past few years have been prolific in the description of new DCM-related *TTN* mutations. A comprehensive cohort study by Herman et al. (16) on 312 DCM patients reported *TTN* truncating mutations to be the cause of DCM in 25 and 18% of familial DCM and sporadic cases, respectively. *TTN* truncating mutations found in subjects with DCM were overrepresented in the A-band region and were absent from the Z-disk and M-band regions. Interestingly, *TTN* truncation variants were also present in up to 2% of the control population, but the control subjects were less enriched for the A-band region of TTN including the Z-band variants. A recent study by Pugh et al. (36) confirmed the presence of truncating variants in the general population (1.65%) and demonstrated that truncating variants located in the A-band are more common in patients with DCM compared with controls. The rate of *TTN* truncating variants found by Pugh et al., in the DCM cohort was ~14%. In addition, a reduced frequency of variants in the I-band was identified in probands compared with controls, whereas no differences were detected in the Z and M bands.

The *TTN* gene has also been evaluated in the European Atlas study of 639 patients with sporadic or familial DCM by NGS. Mutations in *TTN* were identified in 19% of familial and 11% of sporadic cases (37). Noteworthy, 44% of patients with a truncating *TTN* variant also presented an additional known disease-causing variant in at least one other gene involved in the pathogenesis of DCM; thus in these cases, the *TTN* variant may not be the only contributor leading to the pathogenesis of DCM (37).

A large study recently compared the burden of rare *TTN* variants across five cohorts of healthy volunteers, participants in the Framingham Heart Study, participants in the Jackson Heart Study, cohort of unselected ambulatory patients with DCM, and end-stage DCM cases. The authors confirmed that *TTN* truncations were not uniformly distributed within and between study groups, being more common in patients with DCM (22%), but with a rate in the healthy volunteers ranging between 1 and 2.9% (28). The *TTN* truncation variants in the DCM cohort were located predominantly in the A-band, as already described in previous studies mentioned above (16, 36).

**Figure 3:**
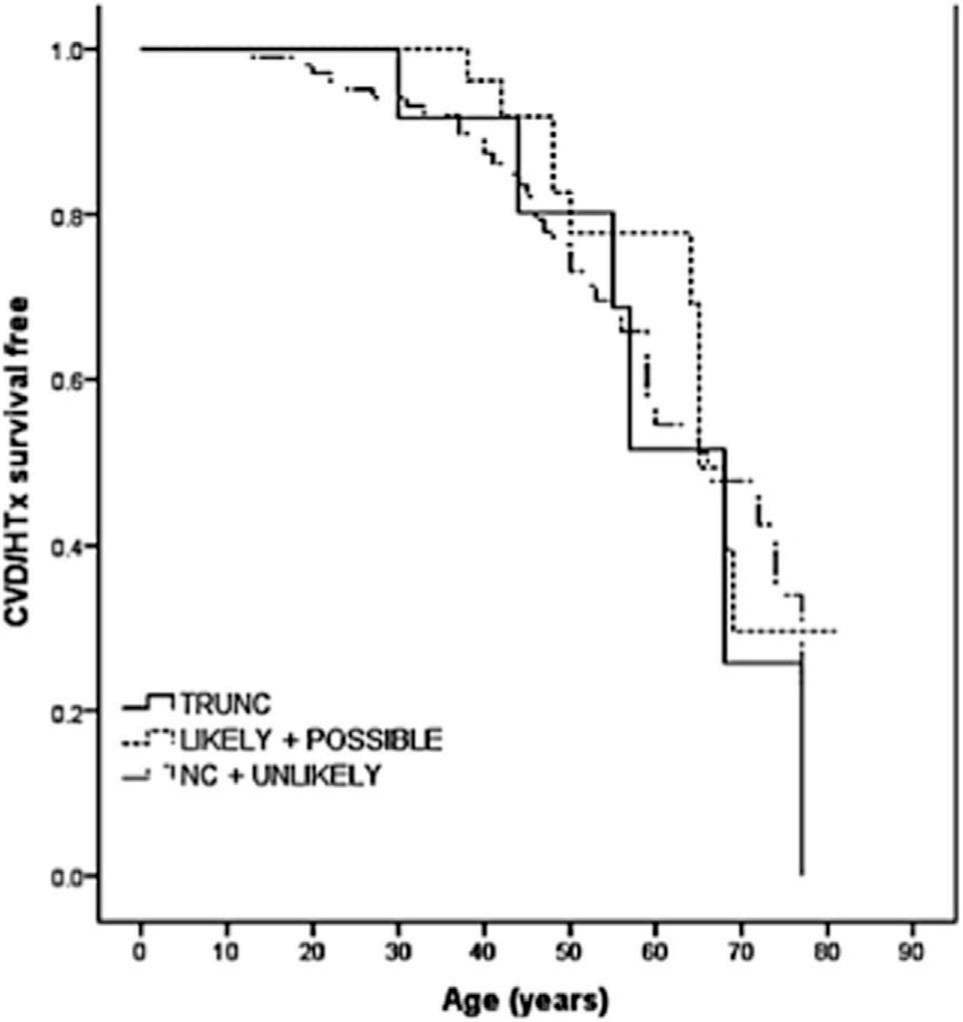
**Long-term survival curves in *TTN* variant carriers**. Kaplan–Meier event-free survival for cardiovascular death (CVD) or heart transplantation (HTx) based on *TTN* variant categories: truncations (TRUNC); “likely” and “possibly” missense variants; non-carriers (NC), and “unlikely” with lack of cosegregation. *TTN* indicates titin gene (from Begay et al., with permission) (38).

The role of *TTN* truncation mutations in the pathogenesis of DCM has been largely recognized. However, the high prevalence of missense variants and the potential modifier effects make it difficult to elucidate the effective role of *TTN* missense variants in DCM. Some of these variants are proposed to be pathogenic, but other variants are of unknown significance (VUS). In order to address this challenge, a recent multicenter study sequenced the *TTN* gene in a cohort of 147 DCM patients (38). In this cohort, 13 *TTN* truncating variants had previously been reported (16), and 348 missense variants were filtered by bioinformatic algorithms resulting in 44 out of 348 (involving 37 probands) classified as “severe” or *likely* pathogenic. Among the nine families with *TTN* variants classified as “severe,” five were considered false positives due to discordant cosegregation analysis among affected relatives, whereas four families had “severe” *TTN* variants that cosegregated with the DCM phenotype. The remaining 28 probands harbored “severe” variants that could not be assessed by cosegregation (*possibly* pathogenic). Furthermore, the outcome of *TTN* missense variants carriers did not differ significantly from the other DCM patients (Figure 3). Interestingly, the distribution of the *likely* and *possibly TTN* severe missense variants across TTN domains was again non-random and was overrepresented in the A-band region of TTN. Specifically, variants were overrepresented in the C-zone of the A-band, which consists of a super repeat of 11 immunoglobulin-like domains and Fn-III domains shown to bind to MyBP-C and subfragment myosin-1, and is essential for the length dependency of force development and calcium sensitivity (39). Therefore, although the real impact of *TTN* missense variants in the pathogenesis of DCM is still unclear, the clustering of variants in the A-band in DCM may suggest that some A-band missense variants may have a functional detrimental effect on contractility and should be further investigated.

## Titin in Other Forms of Cardiomyopathy

### Hypertrophic Cardiomyopathy

Hypertrophic cardiomyopathy is a common and inherited cardiomyopathy with a prevalence of 1 in 500 (40). HCM presents as an unexplained LV hypertrophy, myocardial disarray, and fibrosis that translate in increased risk of life-threatening ventricular arrhythmias, sudden cardiac death, and an increased life-long risk of heart failure (41, 42). In the majority of cases, HCM has an autosomal dominant trait and mutations in at least 11 different genes. These genes encode for sarcomeric proteins that are responsible for 50–65% of familial cases (9). While *TTN* truncation mutations are common in DCM, there is evidence that *TTN* truncations are rare in the HCM phenotype, with a frequency similar to control populations (16). Using high-throughout sequencing in 142 HCM probands, Lopes et al. found 219 *TTN* rare variants with 209 being novel missense variants (43). However, this cohort of individuals potentially had a sarcomeric gene mutation that likely caused HCM, and the actual pathogenic role of these *TTN* variants in unknown.

### Restrictive Cardiomyopathy

Restrictive cardiomyopathy is a very rare form of cardiomyopathy, characterized by preserved biventricular systolic function and a restrictive physiology determining an impaired LV filling despite normal cavity size and frequently normal wall thickness. RCM can be secondary to idiopathic or system disease. It is believed that a significant proportion of RCM cases are genetically determined (42). The pattern of inheritance can be autosomal dominant, autosomal recessive, or x-linked (44). The overall prognosis of RCM is poor, usually resulting in progressive biventricular heart failure with a high mortality rate in the absence of heart transplantation. Interestingly, RCM overlaps in clinical features with HCM (42). Recently, a study using linkage analysis that reported a *TTN* missense variant (*TTN*: c.22862A>G) cosegregating with RCM in six affected individuals of a family. The most common genes were excluded due to lack of complete cosegregation. Interestingly, some healthy individuals also harbored the *TTN* missense variant resulting in an incomplete penetrance (44). The identification of a rare missense variant in *TTN* cosegregating with the RCM disease phenotype suggests that *TTN* is a novel disease-causing gene for RCM.

### Arrhythmogenic Right Ventricular Cardiomyopathy

Arrhythmogenic right ventricular cardiomyopathy is considered to be a genetic disease (30–50% of cases) mainly with autosomal dominant pattern of inheritance (45). ARVC is characterized by fibrofatty replacement of the myocardium, predominantly of the right ventricle, although the left ventricle can also be involved. Typical symptoms include palpitations, cardiac syncope, and cardiac arrest due to ventricular arrhythmias. Heart failure may develop later in life as a result of this disease (46). One study by Taylor et al. in which the investigators analyzed by direct sequencing of 312 exons of *TTN* (311 expressing TTN protein) found *TTN* mutations to be associated with the ARVC phenotype (47). Among seven different probands with an ARVC phenotype, eight *TTN* rare variants (two *TTN* variants present in one proband) were identified (47). In addition to this study, another investigation by Brun et al. compared the clinical outcomes of ARVC patients with *TTN* mutations, desmosomal mutations, and patients with no identifiable mutation (non-carriers) (45). In this study, 13% of *TTN* rare variants were accounted for in their population of subjects. Among the 67 ARVC affected patients (39 ARVC families), 11 harbored rare *TTN* variants and 8 desmosomal genes variants. The *TTN* carriers had increased supraventricular arrhythmias, and conduction disease compared with non-carriers (45), while desmosomal gene variant carriers had the worse prognosis. In conclusion, these studies suggest that *TTN* mutations can cause ARVC and *TTN* mutation carriers have distinct clinical features and outcomes.

## Titin as a Gene Modifier

*TTN* variants are very frequent; of them, pathogenic mutations are relatively rare and most variants are probably benign. However, a portion of these variants could have a *modifier* gene effect. For instance, *TTN* has been proposed as a modifier gene in combination with the Lamin A/C (*LMNA*) gene (48, 49). A *modifier* gene is not the causal gene, but it may affect the phenotypic expression (50). In a study by Roncarati et al., the authors reported a *TTN* missense mutation modifying the DCM phenotype primarily caused by a *LMNA* mutation. The authors analyzed 41 Italian patients using whole exome sequencing (WES). Fourteen individuals harbored *LMNA*: c.656A>C mutation, and of those five also carried a novel *TTN* missense mutation (*TTN*: c.14563C>T) as well (48). *LMNA* gene mutations are known to be causative of a specific phenotype expression of DCM (51). According to Taylor et al., patients carrying a *LMNA* mutation show a poor prognosis and experience high event-rates compared with non-carriers of a *LMNA* mutation (52). Upholding the structure of the nucleus, chromatin arrangement, and gene expression is encoded by the *LMNA* gene for the A-type lamins (53). In a study by Roncarati et al., the presence of the *TTN* variant and the *LMNA* mutation carriers modified DCM patients’ clinical course and disease severity, with double heterozygotes requiring earlier heart transplantation (four individuals) compared with those only harboring the *LMNA* mutation alone. Furthermore, histological studies showed more evidence that double heterozygote individuals had worse outcomes on a cellular level (48). In conclusion, this study suggests a modifier role of *TTN* variants that contribute to the complexity of the DCM phenotype.

## Clinical Assessment of Titin Variants

Titin has been known to be cause a DCM phenotype for many years; however, the systematic analysis and the complete meaning of its contribution to DCM have been precluded by its giant size and sequencing technical limitations (54). As discussed earlier, using NGS, Herman et al. found that heterozygous mutations truncating the full-length TTN are the most common causes of DCM; occurring in ~25% of familial cases of DCM and 18% of sporadic cases. However, *TTN* truncating variants were also found in ~2% of healthy controls (16, 55), raising concern about the correct clinical interpretations of *TTN* variants. The finding of a *TTN* truncating variant in a patient before the onset of clinical manifestation of disease thus requires further in-depth analysis to support pathogenicity (9). Additional factors, such as band location and PSI score, might help to differentiate pathogenic truncation mutations from benign variants (28, 36). This is of particular importance considering that most DCM patients present late in the course of the disease (advanced disease presenting with heart failure or sudden cardiac death), while the early detection of asymptomatic DCM might be critical to enable early intervention that may prevent the progression to advanced disease (56). Moreover, *TTN* truncation variants may be found in association with other disease-related genes, increasing the concerns about the actual role of some *TTN* mutations (37).

Analysis of a large number of genes has led to the identification of sequence VUS. These VUS are one of the main challenges of NGS, because cardiologists and clinical geneticists are faced with uncertainty of the clinical meaning of VUS findings (57).

To date indeed, a large number of identified *TTN* truncating variants are still classified as VUS, and the high prevalence of missense variants in *TTN*, and their potential modifier roles make interpretation difficult in both research and the clinical settings. The location of *TTN* truncating variants can contribute to a better definition of genetic findings, because as already mentioned, *TTN* truncating variants associated with DCM are located predominantly in the A-band (16, 38). The availability of multiple family members to test for cosegregation with disease, the absence in population databases (ClinVar, ExAC, 1000 Genomes Project, and NHLBI Exome Sequencing Project), prediction software (PolyPhen, SIFT, GERP), and functional data also add in the understanding of classifying the pathogenicity of *TTN* variants (9). Most importantly, NGS has to be considered a diagnostic test in development and testing results need to be interpreted cautiously in close collaboration between bioformaticians, cardiologists, molecular biologists, and clinical geneticists preferably in expert centers. Many novel variants identified by NGS and classified as VUS present as an inconclusive test result, pending further evidence (57).

**Figure 4:**
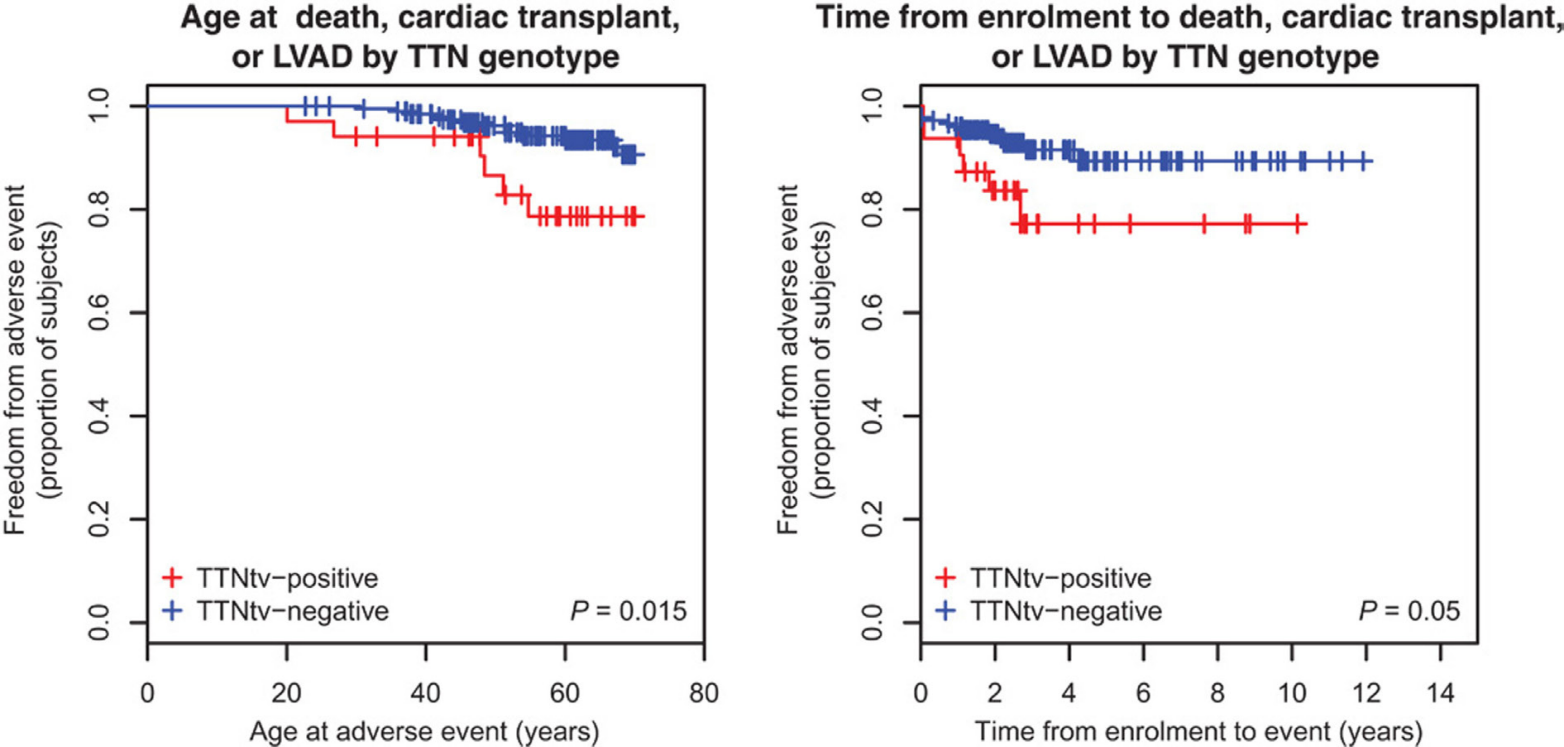
**Survival of *TTN* truncation carriers**. Patients carriers of a *TTN* truncation variant (TTNtv) had a worse clinical outcome when considering the age of adverse event (death, cardiac transplant or left ventricular assisted device) (*P* = 0.015). They also had a worse clinical outcome when considering the time of event from enrollment (from Roberts et al., with permission) (28).

Once the pathogenic effect has been defined, another concern is the variability in phenotype expression based on the presence and type of *TTN* mutation variants. Roberts et al. found more severe impaired LV function, lower stroke volume, and thinner LV walls in *TTN* truncating than in *TTN* truncating negative DCM patients (Figure 4) (28). In this cohort, the *TTN* genotype independently predicted phenotype severity. Furthermore, TTN truncating positive patients more frequently suffer from sustained ventricular tachycardia (28). In the future, if larger prospective studies confirm these findings, *TTN* mutations might influence the decision-making process for the selection of candidates to an implantable cardioverter defibrillator (ICD) implantation, such as in other cardiomyopathies at high risk of life-threatening arrhythmias (58).

Mutations in *TTN* and other proteins affecting *TTN* splicing are associated with the development of DCM, but these mechanisms are still not completely understood (59). Variable isoform expression and *TTN* splicing have become of great importance in DCM, and are associated with decreasing passive stiffness and increasing chamber compliance (26). Both mechanisms might be important in the process of DCM in connection to *TTN* mutations. By genetic approaches or by splicing or posttranslational modifications *TTN* appears to be a target for future therapeutic interventions (9).

Regarding the universe of *TTN* missense variants, the situation is even more challenging because *TTN* missense variants are very common and their real meaning is still unknown. A recent study demonstrated that missense variants did not correlate with the clinical measures of disease severity or progression and indicated that the DCM phenotype caused by *TTN* missense variants are not distinguishable from other types of DCM (Figure 3). According to the authors, *TTN* rare missense mutations should not be currently interpreted as disease-causing in most situations (38). Nevertheless, there is some interesting evidence that *TTN* missense mutations may have a modifier role leading to a greater severity of cardiomyopathy (17, 48). In the future, a better understanding of the *TTN* missense variants in DCM will be elucidated with large-scale *TTN* sequencing and functional investigations on *TTN* variant domains.

Finally, despite the recent advances in genetic studies and in the understanding of the different effects of specific gene mutations in the pathogenesis of DCM, the clinical approach to diagnosing cardiomyopathy affected families remains largely based on the general recommendations for heart failure management, familiar screening programs, and systematic follow-up. The continuous improvement in technologies, such as the increasing evidence concerning the clinical expression of different gene variants might lead in the future to an individualized clinical approach to identifying carriers of different mutations.

## Conclusion

Titin is the largest protein in striated muscle. *TTN* variants have been shown to cause the following cardiac diseases: DCM, RCM, HCM, and ARVC. The advancement of NGS has allowed researchers to analyze the whole *TTN* gene, which has revealed the leading role of this gene in DCM. Challenges are the high genetic variability of the gene, the large number of missense and truncation variants found in control populations, and the criteria for clinical diagnosis of many variants demand individualized clinical diagnosis platforms for *TTN* carriers. Future studies will clarify whether the early identification of *TTN*-related cardiomyopathies might positively influence the natural history of disease by the early initiation of therapeutic management.

## Author Contributions

All authors have contributed significantly, read, and approved the manuscript. In particular, MG, RB, and GM: drafting of the manuscript; GS, MT, HG, and LM: revising critically the manuscript for important intellectual content.

## Conflict of Interest Statement

The authors declare that the research was conducted in the absence of any commercial or financial relationships that could be construed as a potential conflict of interest.
